# Nationwide implementation of personalized outcomes forecasts to support physical therapists in treating patients with intermittent claudication: Protocol for an interrupted time series study

**DOI:** 10.1371/journal.pone.0288511

**Published:** 2023-07-31

**Authors:** Anneroos Sinnige, Andrew Kittelson, Katrien M. Rutgers, Laura H. M. Marcellis, Philip J. van der Wees, Joep A. W. Teijink, Thomas J. Hoogeboom

**Affiliations:** 1 Department of Vascular Surgery, Catharina Hospital, Eindhoven, the Netherlands; 2 CAPHRI Research School, Maastricht University Medical Centre, Maastricht, the Netherlands; 3 School of Physical Therapy and Rehabilitation Science, University of Montana, Missoula, MT, United States of America; 4 Physical Therapy Sciences, Program in Clinical Health Sciences, University Medical Center Utrecht, Utrecht University, Utrecht, The Netherlands; 5 IQ Healthcare, Radboud Institute for Health Sciences, Radboud University Medical Center, Nijmegen, the Netherlands; UNITED STATES

## Abstract

**Introduction:**

Shared decision-making is the cornerstone of patient-centered care. However, evidence suggests that the application of shared decision-making in physical therapy practice is limited. To elicit shared decision-making and thereby potentially improve patient outcomes for patients with intermittent claudication, we developed a decision support system. This decision support system provides personalized outcomes forecasts that visualize the estimated walking distance of an individual patient. We hypothesize that personalized outcomes forecasts can support physical therapists in personalizing care to the needs and priorities of the individual patient to improve therapy outcomes.

**Research objectives:**

The primary aim is to evaluate the impact of personalized outcomes forecasts for patients with intermittent claudication to optimize personalized treatment. Second, this study aims to evaluate the process of implementation.

**Methods:**

This study uses a prospective interrupted time series (ITS) design. Participating physical therapists are divided into four clusters. Every month of the study period, a new cluster will be invited to begin using the decision support system. We aim to include data of 11,250 newly referred patients for physical therapy treatment. All therapists associated with a network of specialized therapists (Chronic CareNet) and patients treated by these therapists are eligible to participate. The decision support system, called the *KomPas*, makes use of personalized outcomes forecasts, which visualize the estimated outcome of supervised exercise therapy for an individual patient with intermittent claudication. Personalized outcomes forecasts are developed using a neighbors-based approach that selects patients similar to the index patient (a.k.a. neighbors) from a large database. Outcomes to evaluate impact of implementation are patients’ functional and maximal walking distance, quality of life and shared decision-making. Process evaluation will be measured in terms of utilization efficacy, including the outcomes dropout rate and reasons to (not) use the personalized outcomes forecasts. Data will be routinely collected through two online systems: the Chronic CareNet Quality system, and the website logs of the decision support system. Additionally, observations and semi-structured interviews will be conducted with a small subset of therapists.

**Ethics:**

Formal medical ethical approval by the Medical Research Ethics Committees United ‘MEC-U’ was not required for this study under Dutch law (reference number 2020–6250).

## Introduction

Treatment guidelines for patients with peripheral arterial disease encourage physical therapists to make shared decisions [1,2]. Shared decision-making is an approach for physical therapists to integrate evidence-based knowledge with patients’ experiences and preferences [3,4]. The shared decision-making process consists of three stages: 1) team talk, preparing the patients for collaboration; 2) option talk, exchanging information about treatment options; and 3) decision talk, affirming and implementing the decision or plan [5,6]. This patient-centered approach is associated with improved patient outcomes.5 Although shared decision-making is currently considered the norm, evidence suggests that its application in daily physical therapist practice is very limited [7–9]. A potential explanation for the lack of shared decision-making in daily practice is the lack of available and useful decision aids [10,11]. Therefore, to elicit shared decision-making and thereby improve outcomes for patients with intermittent claudication, we developed a decision support system [12].

Intermittent claudication is the most common symptom of peripheral arterial disease, caused by atherosclerotic narrowing of the arteries in the lower extremity. Intermittent claudication is defined as walking induced discomfort and pain in the hip and leg muscles that typically disappears after a brief rest. The recommended first treatment for these patients is supervised exercise therapy (SET) and lifestyle guidance, provided by physical therapists [1,2]. The decision support system that we developed aims to promote personalized treatment for this patient population and to elicit shared decision-making by providing therapists and patients insight into an individual’s personal prognosis. Insight into an individual’s personal prognosis is created by the use of personalized outcomes forecasts, which visualize estimated improvements in walking distance over the course of SET. These estimates are based on historical outcomes data of patients similar to the index patient, and have been described previously (see methods section) [12–14]. Personalized outcomes forecasts are intended to support physical therapists in their clinical reasoning and in shared decision-making processes. We also hypothesize these forecasts will help therapists in personalizing care to the needs and priorities of the individual and thereby potentially improve patient outcomes.

The use of this decision support system with personalized outcomes forecasts is a novel approach in the conservative treatment of patients with intermittent claudication. Accordingly, the impact of utilizing efficacy personalized outcomes forecasts by physical therapists is unknown. The decision support system is now being implemented within the Chronic CareNet network of specialized therapists in the Netherlands [15], which offers an opportunity to study its potential merits. In this protocol, we describe the methods of implementation and the study design to assess impact and process evaluation.

### Research objectives

The primary aim of this study is to evaluate the impact of implementing personalized outcomes forecasts (i.e., KomPas) in the daily practice of physical therapists working with patients with intermittent claudication to optimize personalized treatment on the following outcomes: walking distance, quality of life, and shared decision-making, as compared to usual care. A summary of specific research questions can be found in Table 1.

**Table 1:** 

Specific research question	Outcome measure	Measurement tool:	Data routinely collected:	Data collection:	Sample size:
1.1 What is the effect of implementing the decision support system in daily practice on the functional walking distance?	Functional walking distance	Treadmill test	Yes	Export from the Quality system database and Personalized outcomes forecasts	11,250
1.2 What is the effect of implementing the decision support system in daily practice on the maximal walking distance?	Maximal walking distance	Treadmill test	Yes	Export from the Quality system database and Personalized outcomes forecasts	11,250
1.3 What is the effect of implementing the decision support system in daily practice on the Quality of Life?	Quality of life	VascuQoL-6	Yes	Export from the Quality system database and Personalized outcomes forecasts	11,250
1.4 What is the effect of the decision support system on shared decision-making during the initial visit?	Shared decision-making	Option 5	No	Participatory observations	30

The secondary aim of this study is to examine utilization efficacy of personalized outcomes forecasts and evaluate the implementation process (see Table 2 for a summary of secondary research questions).

**Table 2:** 

Specific research question	Explanation	Measurement tool?	Data routinely collected?	Data collection?	Sample size
2.1 “What percentage of the eligible physical therapists use the decision support system during the study period?”	Use of the decision support system	Website logs	Yes	Personalized outcomes forecasts export	11,250
2.2 Why did physical therapists (not) use the decision support system during the study period?	Reasons for (not) using it	Qualitative data	No	Semi-structured interviews	15–20
2.3 What is the effect of the decision support system on dropout rate of patients during SET?	Dropout rate	Website logs	Yes	Personalized outcomes forecasts export	11,250

## Methods

### Study design and setting

**Fig 1:**
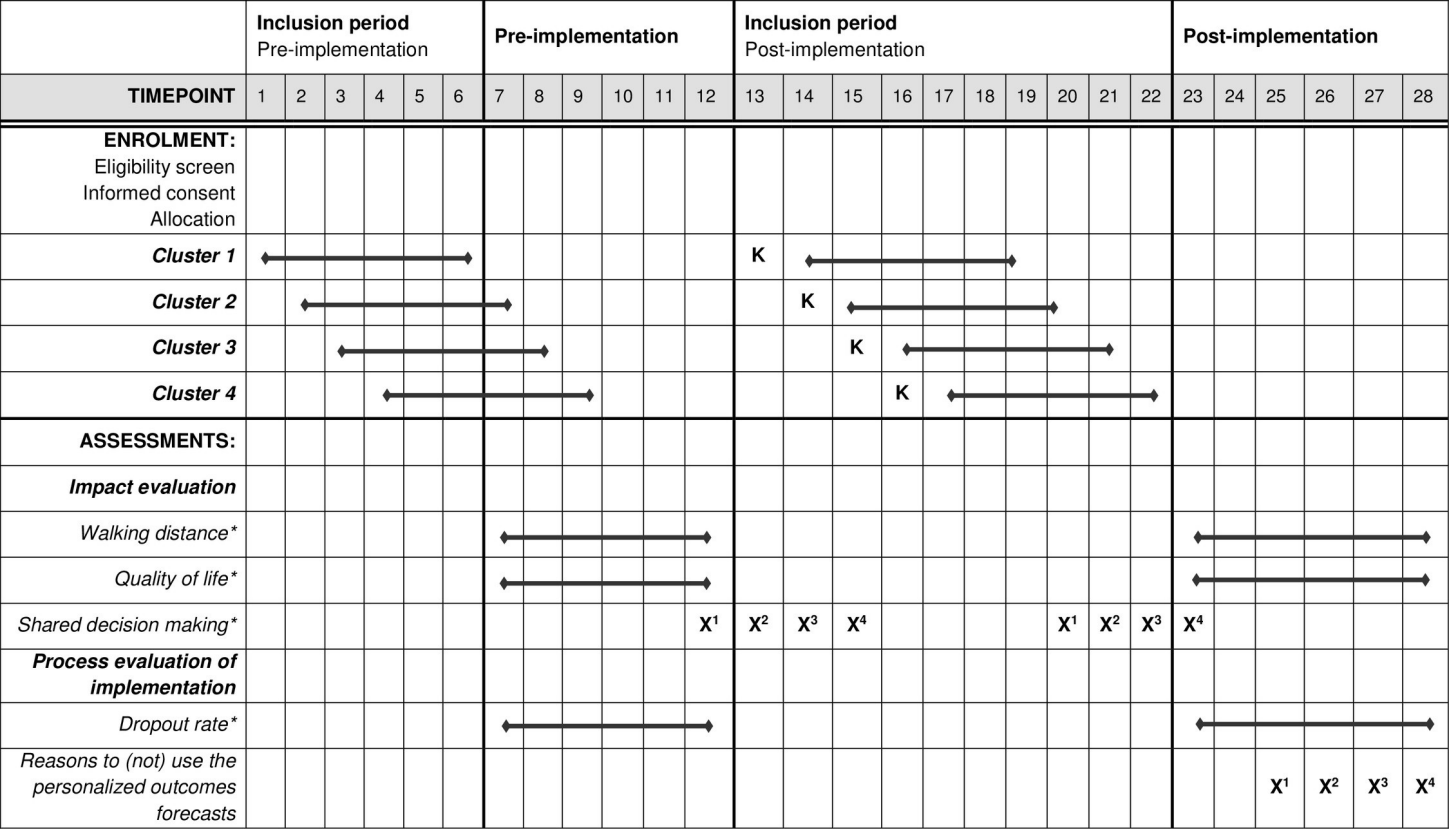
SPIRIT figure. The figure shows the different time periods and division into clusters of the trial including the data collection time points. K = Implementation KomPas in the specific clusters. * = Data which is routinely collected through two by physical therapists as part of daily practice. Routine data is collected through two online systems: The Chronic CareNet Quality system, and the website logs of the decision support system. X = Data collection point of non-routine data for the specific clusters.

To answer our research questions, we will make use of a prospective interrupted time series (ITS) design with a parallel process evaluation (‘‘Netherlands Trial Register” registration number: NL8838) [16]. The ITS study design is useful to examine whether an intervention has an effect additional to a possible underlying secondary trend [17]. In this design, enrollment of physical therapists will take place in clusters, based on geographical region. Every month, a new cluster will be enrolled and physical therapists will receive an email with the invitation to start using the personalized outcomes forecasts in the treatment of patients with intermittent claudication (see Fig 1 for the complete timeline). Data collection is divided into a pre-implementation (i.e., control) period and a post-implementation (i.e., experimental) period, within each region (see Fig 1). Patients seen for care during a region’s pre-implementation period will receive usual care as KomPas is not yet available. Patients seen during the post-implementation period will have access to KomPas and associated personalized outcomes forecasts via interactions with physical therapists. At the level of the region, pre- and post-implementation periods last one year and are divided into the first six months comprising the inclusion period and the second six months comprising the follow-up period (i.e. data collection period). This was done to ensure that all patients enrolled during the pre-implementation (i.e., control) period receive standard care, uninfluenced by personalized outcomes forecasts.

### Participants and context

The personalized outcomes forecasts have been specifically developed to use in the treatment of patients with intermittent claudication by physical therapists associated with Chronic CareNet. Chronic CareNet is a network of therapists specialized in the treatment of patients with chronic non-communicable diseases, including intermittent claudication [15]. In the Netherlands, SET is only completely reimbursed by basic healthcare insurance if the therapy is provided by Chronic CareNet therapists. Therefore, the network has substantial coverage in this patient population.

All physical therapists affiliated with Chronic CareNet participate in routine data collection through the Chronic CareNet Quality system. This Quality system has been set up in collaboration with the National Database of the Royal Dutch Society for Physical Therapy (KNGF), which has gathered data of all patients with intermittent claudication treated with SET since 2015 [15]. Data in the Quality system are routinely collected by physical therapists as part of daily practice. Patients will be asked to provide general informed consent for the use of their data by Chronic CareNet for research purposes. For patients that provide informed consent, pseudo-anonymized data will be gathered through the electronic health records. All patients referred to a Chronic CareNet therapist, which specialize in the treatment of intermittent claudication, are eligible to participate in this study. Exclusion criteria are not applicable due to the pragmatic nature of this study. According to the SET protocol, a course of therapy lasts 12 months and includes five measurement time points: at baseline (initiation of treatment) and every three months thereafter. For this study, the six month follow up will be used as end point, since the first six months encompass care planning, as well as the period of the greatest expected treatment response. For semi-structured interviews examining therapist use (or lack of use) of KomPas, we will purposefully sample therapists based on their level of adoption of the personalized outcomes forecasts in their daily practice [18,19]. Briefly, we will categorize therapists into tertiles based on the rate of utilization efficacy of the KomPas tool, and we will seek to recruit interviewees from each of the categories (i.e., high, medium, and low adoptors).

### Decision support system

**Fig 2:**
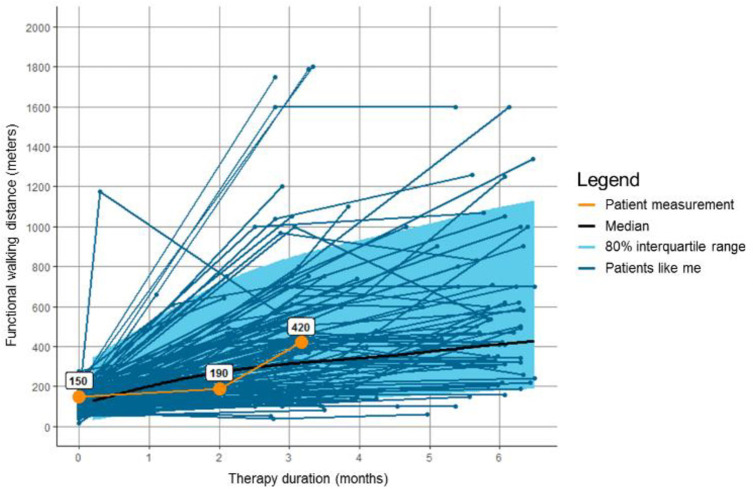
Personalized outcome forecasts, visualizing a patient’s individual prognosis of supervised exercise therapy, based on historic data of patients similar to the index patient.

The decision support system makes use of personalized outcomes forecasts. These forecasts visualize the estimated outcome of SET, walking distance on a standardized treadmill test, for an individual patient with intermittent claudication (see Fig 2) [12–14]. A common known example of such forecasts are the reference charts used for monitoring infant growth that plot individual growth against growth of similarly aged infants [14]. Personalized outcomes forecasts are developed using a neighbors-based approach to create the individual forecasts using historical outcomes data. This method selects patients similar to the index patient (a.k.a. “neighbors” of the index patient) from a large database, based on age, sex, smoking history (pack years), Body Mass Index (BMI), motivation for behavior change, and baseline functional walking distance. The actual outcomes data of these similar patients are then used to create an individual forecast [12–14]. Personalized outcomes forecasts are incorporated in an online decision support system, called the *KomPas* (i.e., “Compass,” in Dutch). The *KomPas* automatically plots the estimated therapy outcome over time, thereby making personalized outcomes forecasts easily accessible and interpretable [12]. The decision support system was developed based on feedback from daily practice and the first use was investigated in both a pilot study and an explorative study. Results of these studies were used to optimize the tool and the implementation plan [20].

### Implementation of personalized outcomes forecasts

Personalized outcomes forecasts will be implemented according to the ITS design in four study clusters at equal intervals of one month. In this study, implementation involves making the personalized outcomes forecasts available for therapists to use in daily practice. Chronic CareNet comprises 55 regions (based on ZIP codes) in the Netherlands, four of which already use the personalized outcomes forecasts in the context of the pilot study and explorative study [20]. Each study cluster contains 12 to 14 regions, the number of therapists per region ranges from 25 to 100, resulting in clusters that comprise 400–600 therapists. After implementation, a six-month inclusion period starts (see Fig 1). Use of the personalized outcomes forecasts is not mandatory, but will be promoted in several ways, including reminder emails, newsletters and online webinars. Furthermore, during this inclusion period, therapists can gain experience using the tool. To promote therapists’ use of personalized outcomes forecasts, three online trainings will be made available: a basic training and two in-depth trainings. The basic training will contain all necessary information to start using the personalized outcomes forecasts in daily practice. For example, this training covers how to create a personalized outcomes forecast for an individual patient and how to interpret this forecast. This basic training is mandatory to complete before therapists can start using the personalized outcomes forecasts in daily practice. Two additional in-depth e-learnings will be made available to increase knowledge on personalized outcomes forecasts. These in-depth e-learnings include videos of therapists and patients using the personalized outcomes forecasts during treatment sessions. The aim of these e-learnings is to promote therapist confidence and fluency with daily use of the forecasts.

### Data collection

#### Impact evaluation

The outcomes to determine the impact of the implementation are: 1) functional and maximal walking distance (assessed using a standardized walking test), 2) quality of life (assessed using a questionnaire) and 3) shared decision-making (Table 1).

*Functional and maximal walking distance*. Data regarding functional and maximal walking distance will be gathered through the Chronic CareNet Quality system and the personalized outcomes forecasts website. Data gathered through the personalized outcomes forecasts website are always data generated through use of the outcomes forecasts. Functional and maximal walking distance are measured by physical therapists as part of clinical daily practice, using a standardized treadmill test (i.e., Gardner Skinner protocol) [21]. The test protocol describes a speed of 3.2 km/h with an incline of 0% at start, increasing 2% every 2 minutes. Maximal walking distance is defined as the walking distance where intolerable claudication pain forces a patient to stop. The functional walking distance is defined as the distance at which the patient would prefer to stop walking due to the pain. This treadmill-based measurement is recommended by the Dutch treatment guideline and has demonstrated excellent reliability and construct validity in previous research [22,23].

*Quality of life*. Data regarding quality of life will also be gathered through the Chronic CareNet Quality system and the personalized outcomes forecasts website. Quality of life will be assessed using the Vascular Quality of Life Questionnaire-6 (VascuQol-6). This questionnaire is the shortened version of the VascuQol-25 and is used by physical therapists as part of clinical daily practice. Questions are related to a patient’s daily activity, current symptoms, pain, and social well-being [24].

*Shared decision-making*. Shared decision-making will be measured by applying the OPTION-5 scale to audio or video recordings of clinical observations. The OPTION-5 is based on a previous 12-item measure, but more specific to the construct of shared decision-making [25]. Assessment of the recordings will be performed by specifically trained physical therapists experienced in using the personalized outcomes forecasts. A random subgroup of physical therapists will be invited by mail and phone to participate in these observations. All therapists who receive a new referral of a patient for SET are eligible to participate.

#### Process evaluation of implementation

The secondary aim of this study—to assess the success of implementation—will be measured in terms of uptake, or utilization efficacy (Table 2). This represents the extent to which personalized outcomes forecasts are likely to be used [16]. This will be measured using dropout rate and reasons to (not) use the personalized outcomes forecasts.

*Dropout rate*. Dropout rate will be extracted from the website logs and operationalized as the percentage of patients that terminate therapy before the treatment goal is achieved. Termination of therapy is indicated by the therapist in the health record. We will also assess missing value (i.e., the percentage of missing measurements at 3 and 6 months).

*Reasons to (not) use the personalized outcomes forecasts*. **Semi-structured interviews will be performed with therapists who have use the personalized outcomes forecast with at least one patient, to obtain reasons for (not) using the forecasts.** The purpose is to uncover ways to improve the uptake of personalized outcomes forecasts or other electronic decision support systems in this patient population. Semi-structured interviews will be conducted once at 6 months follow-up.

### Sample size

Different samples will be recruited to answer the different research questions (see Tables 1 and 2). For several research questions, data will be collected through the Quality system itself. We estimate the Quality system accrues data at a rate of approximately 15,000 new patients with intermittent claudication annually. For the pre-implementation period, we will use the data routinely collected through the Quality system. Thus, we estimate that the six-month “control” period will ultimately include data for 7,500 patients in total. For the post-implementation period, we expect that 50% of all therapists affiliated with Chronic CareNet will start using the personalized outcomes forecasts in daily practice. This is based on preliminary work (unpublished data). Thus, we expect to include 3,750 patients for the post-implementation period. In total, we expect to include 11,250 patients in the analysis examining effectiveness outcomes. For the examination of shared decision-making, we will aim to obtain 30 observations, according to previous studies that have used similar measurements [26,27]. Based on previous pilot testing (unpublished data), we will account for possible attrition of 20–30%. Therefore, we aim to include 36 to 39 (30*1.20–1.30 = 36–39) therapists in total. For the semi-structured interviews with therapists to measure reasons for (not) using the tool, data will be sampled until saturation is reached. Data saturation is expected to be reached around 15 to 20 therapists in accordance with previous research [20].

### Data analysis

Statistical analysis will be performed by using IBM SPSS Statistics for Windows, version 28. Baseline participant characteristics will be summarized using descriptive statistics. Continuous variables will be reported in means and standard deviation (SD) and categorical variables will be reported as frequencies and percentages.

#### Impact evaluation

Outcomes to evaluate the impact of implementation are maximal and functional walking distance (measured by standardized treadmill test), quality of life, and shared decision-making. Outcomes will be operationalized as the change from baseline to a six-month follow up. We will perform an intention to treat analysis with the data exported from the Quality system database, since this database includes data of all patients treated with SET, with or without use of the personalized outcomes forecasts. A per protocol analysis will be perform using the data exported from the personalized outcomes forecasts. To compare pre- and post-implementation, segmented regression analysis will be performed. Segmented regression analysis is a commonly used method in ITS designs that accounts for possible changes in level and trend as result of implementation of a certain intervention [28]. Serial autoregressive correlation due to repeated measures will be checked for each outcome measure using the Durbin-Watson test [29]. If no significant autocorrelation is present, a simple time series regression will be used. In case of significant autocorrelation, we will adjust for effects as required. Graphical representation of results will be used to visually inspect change in outcome over time [30]. We will also visually check whether the profile(s) over time in the clusters suggest non-linear time effects. Also, as an additional analysis, we will investigate the influence of quadratic, cubic, and possibly quartic and quintic polynomials of the ‘time’ variable in the segmented regression. Polynomials of degree five typically capture almost all non-linearity present. We will use fit-statistics such as Akaike’s information criterion to assess whether possibly improved fit outweighs the added complexity of these models. Individual regression coefficients will be combined in meta-analyses to provided overall estimates [31]. To determine the effect size, we will use the minimally important difference, which is available from previous research [32,33].

Shared decision-making will be measured using the OPTION-5 score. OPTION-5 scores will be analyzed using a paired t-test to demonstrate a statistical significant difference in the mean overall score between pre and post measurement. Data will be tested for normality using skewness and kurtosis [34]. If the data are not normally distributed, a Wilcoxon matched-pairs test will be used. An alpha level of 0.05 and a 95% confidence interval will be used.

#### Process evaluation

Outcomes to assess the process evaluation of implementation in terms of utilization efficacy, include dropout rates (documented in the health record) and reasons to (not) use the personalized outcomes forecasts (assessed by semi-structured interviews). The pre- and post-implementation drop-out rate will be compared using segmented regression analyses, as described for impact evaluation. Qualitative data on reasons to (not) use the personalized outcomes forecasts gathered through semi-structured interviews will be described in the overall synthesis of the findings.

### Ethics

Formal medical ethical approval by the Medical Research Ethics Committees United ‘MEC-U’ was not required for this study under Dutch law (reference number 2020–6250). All physical therapists affiliated with Chronic CareNet adhere to the data delivery procedures as this is a participation conditions of the network. In other words, all affiliated therapists agree with the necessary use of data when participating in the network and thus also for the purpose of this study. Patients who receive care via a Chronic CareNet therapist provide consent for the use of their clinical data for research and clinical purposes. This enables a number of critical functions within Chronic CareNet; for example: the storage of patient data and recall of treatment results through visuals in the website, the use of aggregated data for quality improvement and research analyses, and the semi-automated generation of standardized documentation (for example, feedback letters for referring physicians regarding patient progress). Informed consent of patients is collected digitally through the Chronic CareNet website with a digital signature. By signing this informed consent, patients agree to the use of their data for research purposes. Data will be handled confidentially and in accordance to the General Data Protection Regulation (AVG).

Patients and therapists undertaking the shared decision-making observations or semi-structured interviews will be separately consented prior to participation in this part of the project. After formal agreement to participate, therapists receive written information, informed consent forms and self-addressed envelopes by post for both themselves and the patient. For the observations, information is provided to the patient by the therapist. Both therapist and patient will sign two consent forms. All forms will be sent in the self-addressed envelope to the coordinating investigator, who will sign the forms and return one for the patient and one for the therapist.

## Discussion

This protocol describes an Interrupted Time Series (ITS) study examining the implementation of personalized outcomes forecasts (KomPas) for physical therapy treatment of patients with intermittent claudication in the Netherlands. This pragmatic study design and approach (lacking exclusion criteria) aims to overcome selection bias and improve generalizability of study findings [35]. Additionally, the ITS design makes it possible to account for underdlying trends to better isolate the effects of the intervention.

### Limitations

Two limitations should be considered for this study. First, the use of the personalized outcomes forecasts is not mandatory for the physical therapists within the Chronic CareNet network. Therefore, challenges may arise when implementing this (or any) new approach that necessarily affects workflow. Therapists will have to implement this tool into their practice routine and invest time to do so, based on internal motivation. Time pressure is high within the physical therapy profession and additional administrative workload is a barrier for implementation [36]. Although the use of personalized outcomes forecast may seem to cost additional time initially, we believe it may also speed or ease certain aspects of therapist workflow (e.g., care-planning cognitive burden, documentation time). Thus, the KomPas tool may also save time or otherwise reduce friction costs in daily practice. Ultimately, our planned analysis of qualitative data aims to uncover barriers and facilitators to the use of KomPas. To make implementation as successful as possible, good communication on the additional value of using the personalized outcomes forecasts is important. Therapists will also be supported in using the personalized outcomes forecasts. This support includes online training and webinars. Furthermore, therapists have been involved in the development as much as possible to make the personalized outcomes forecasts answer their needs from daily practice. The second limitation is the division of participants into only four clusters with only one month in between start dates of the clusters. Ideally, we could have used more clusters with larger time intervals. However, this would be too time consuming and not possible within the timeframe of the project.

## Conclusion

This planned study aims to improve patient-centered care for patients with intermittent claudication through use of a novel decision support tool that generates personalized outcomes forecasts. The tool has been designed to elicit shared decision-making and aid in the personalization and optimization of treatment.

## Supporting information

S1 Checklist SPIRIT 2013 Checklist: Recommended items to address in a clinical trial protocol and related documents*. (DOC)
